# Active Travel by Built Environment and Lifecycle Stage: Case Study of Osaka Metropolitan Area

**DOI:** 10.3390/ijerph121215027

**Published:** 2015-12-15

**Authors:** E. Owen D. Waygood, Yilin Sun, Laurence Letarte

**Affiliations:** 1 Graduate School of Land Management and Regional Planning (ESAD), Laval University, 2325 des Biblotheques Street, Quebec, QC G1V 0A6, Canada; laurence.letarte.1@ulaval.ca; 2 Department of Civil Engineering and Architecture, Zhejiang University, Zhejiang 310027, China; yilinsun@zju.edu.cn

**Keywords:** built environment, active travel, household lifecycle stage, Japan

## Abstract

Active travel can contribute to physical activity achieved over a day. Previous studies have examined active travel associated with trips in various western countries, but few studies have examined this question for the Asian context. Japan has high levels of cycling, walking and public transport, similar to The Netherlands. Most studies have focused either on children or on adults separately, however, having children in a household will change the travel needs and wants of that household. Thus, here a household lifecycle stage approach is applied. Further, unlike many previous studies, the active travel related to public transport is included. Lastly, further to examining whether the built environment has an influence on the accumulation of active travel minutes, a binary logistic regression examines the built environment’s influence on the World Health Organization’s recommendations of physical activity. The findings suggest that there is a clear distinction between the urbanized centers and the surrounding towns and unurbanized areas. Further, active travel related to public transport trips is larger than pure walking trips. Females and children are more likely to achieve the WHO recommendations. Finally, car ownership is a strong negative influence.

## 1. Introduction

Active travel contributes to physical activity accumulated over a day and is found to more frequent in more urbanized areas (e.g., higher population and service densities, mixed land-use, *etc.*). However, although more frequent, the trips are generally of shorter distances and thus shorter duration. In a society, such as Japan, where commuting by public transport is common, individuals who live in less urbanized areas may thus gain physical activity if they access public transport by walking or cycling (e.g., active travel). Therefore, one question here is *whether there is a difference in active travel by level of urbanization (i.e., the built environment)*. 

Confounding the impact of the built environment are two key considerations: household car ownership and household life-cycle stage. Household car ownership can affect the results in at least two ways. The first relates to the negative effect of owning a car on the likelihood of making all trips by active travel or public transport. The second is that a greater ownership level would act as a proxy for the value or preference put on car use by the household.

The second factor considered here is the household life-cycle stage. As above, there are at least two ways that this may influence the results. The first relates to household needs and desires. A household with small children will not have the same transportation needs and wants as one with older (and likely more independent) children. The second is through the unequal distribution of household type by build environment type. If single and young couples are more likely to locate in central and highly urbanized areas as compared to families with children, then omitting this would result in findings that are perhaps more indicative of the different needs and desires of those households.

Thus, this paper will examine whether there is a difference in active travel by level of the built environment when household car ownership and household life-cycle stage are accounted for. The differences between the areas are put into perspective by recommendations and tools from the World Health Organization (as described below).

### 1.1. Benefits and Evidence of Active Travel

Active travel is any travel where a person must use physical energy to propel themselves such as walking, cycling, skateboarding, or using a wheelchair amongst others. As such, it is most commonly associated with physical health benefits. In a review of evidence to support the inclusion of active travel in health assessments, De Nazelle *et al.* [1] found that there are large individual health benefits to active travellers and smaller population-wide benefits from reductions in air (physical health) and noise pollution (mental health).

Active travel is also one of the three physical activity domains, along with leisure time and occupational activity that contribute to the attainment of the World Health Organization (WHO) global recommendations on physical activity for health [2]. The World Health Organization recommends that young people (aged 5–17 years) should be getting an accumulation of at least 60 minutes of moderate to vigorous physical activity daily [2]. For adults aged 18–64 and for older adults as well, it is recommended that they achieve 150 min of moderate activity over a week, and that the activities should be in bouts of at least 10 min. Thus, to reach the WHO recommendation through transportation, adults should accumulate 30 min of active travel every day (which would suggest 150 min for five equivalent weekdays over a week) where trips are at least 10 min long.

Walking and cycling are certainly the most common ways to achieve active transportation but their contributions to WHO recommendation on physical activity depends on their intensity. Walking at 4 km/h (3 mph) sis considered to be a moderate-intensity physical activity where there is a noticeable increase in heart rate [3]. In order to be considered a vigorous-intensity activity, walking should be done at the very brisk pace of 7.25 km/h (4.5 mph) [3]. In a study designed to evaluate the walking speed of recreational walkers, Murtagh *et al.* [4] showed that adults intuitively walk at a speed that meets the definition of moderate intensity physical activity, and when encourage to walk briskly, most people meet the vigorous intensity threshold.

According to the Compendium of Physical Activities, cycling to and from work, at a self-selected pace, can be considered a vigorous activity, though the same reference also suggests that in order to be considered a vigorous intensity activity, cycling should be done at a pace of at least 16 km/h (10 mph) [3]. Either way, a recent review found numerous physical health benefits of cycling. For young people those benefits included health and functional benefits. For middle-aged and elderly people those benefits included: “improvements of cardiorespiratory fitness and disease risk factor, as well as a significant risk reduction of for all-cause and cancer mortality and for cardiovascular, cancer, and obesity morbidity” [5]. For working-age adults who cycle-commuted there was “improvement in cardiovascular fitness and some improvements in cardiovascular risk factors” [5]. Thus there is some suggestion that cycling would require greater effort than walking, though whether this holds across different cycling cultures is not clear.

Walking and cycling are also key access modes for use in public transport. In a review articles reporting active travel associated with public transport use, a range of 8–33 additional min of active travel was found [6]. All of the papers cited in that review came from Anglo-Saxon countries. In that article, it reported findings from the Bus Association Victoria (of Australia) that public transport users averaged 41 min per day of active travel. Using the 2001 National Household Travel Survey in the USA, Besser and Dannenberg [7] found that public transit users had a mean total of 24.3 min and a median of 19 min. The use of public transport is often found to be higher in wealthy Asian cities such as Singapore, Hong Kong, and Tokyo (average 32.3% of modal share) than what is typically found in the USA (3.4%) and Australia and New Zealand (5.1%) [8]. As public transport works on an economies of density, in such cities, the distances required to reach public transport may be substantially lower than results found in the review by Rissel, Curac, Greenaway and Bauman [6]. In a study of Dutch active travel using the Dutch National Travel Survey (2010–2012) that included walking, cycling, and active travel access to public transport, one could calculate from the findings and methods provided that on average the Dutch population achieved an average of 1.6 min per day through active travel to and from public transport (using the same method, the population average accumulation of active travel min from walking and cycling were roughly 11 and 14 min, respectively) [9]. This may suggest that in dense environments (e.g., the Dutch context *versus* the Anglo-Saxon ones) that support a high service level of public transport, the physical activity associated with public transport use may not be as great. However, it was not clear from the study how many min of active travel were achieved for public transport trips, only the average over the entire population. Thus, one question to resolve would be to know the average active transport time associated with public transport in dense environments where public transport use is common (such as in an Asian context).

Physical health benefits are not the only possible benefits to health accrued through active travel. As well-being is composed of at least three domains: physical, psychological, and social [10]. As relates to psychological well-being, Martin *et al.* [11] found that there were overall psychological benefits to active and public transport commuting in comparison to car commuting. In children’s travel research, children who commuted to school by bicycle were found to have greater activation (e.g., they were more alert) than those who came by car [12], and children who used active transport to school reported more positive emotions than those who used passive modes (e.g., car, bus) [13]. Finally, Gatersleben and Uzzell [14] found that people traveling by active modes were more likely to report that their journey was relaxing as compared to car users.

Social relationships or rather a lack thereof, have a mortality risk on par with tobacco and greater than obesity [15]. As well, social relationships such as social capital (*i.e.*, ones social networks) are important predictors of subjective well-being [16,17]. Sociology work suggests that walking in one’s neighborhood is an important means of building social capital [18]. For children in Japan, walking and more urbanized areas were associated with more frequent occasions where children would see people in general, but perhaps more importantly, with seeing people that they knew while traveling [19]. For adults, Hanibuchi *et al.* [20] did not find a correlation between the walkability of a neighborhood and social capital. Their findings did suggest that the level of urbanization and how old the neighborhood was were positively associated with many social capital variables. In a study that directly examined whether walking (as opposed to walkability) was associated with local sociability in Australia, du Toit *et al.* [21] found a weak positive relationship between the walkability index and a sense of community, which was better explained by transport (utilitarian/ functional) walking than by recreational walking. In that study, respondents were asked about the frequency of nine different types of social interactions with neighbors over the previous month. Leyden [22] found that for Ireland people living in walkable, mixed-use neighborhoods had higher levels of social capital than those living in car-oriented suburbs.

There are negative consequences associated with active travel as well. One study of active travel in China [23] found that there were negative correlations between active travel and certain health outcomes such as cholesterol disorder, and diabetes. In other studies, the negative associations were a potential increase in risk of collision and deeper inhalation of air pollutants on the physical health side (e.g., [1], and a negative correlation between psychological well-being and longer commutes (this of course assumes that a switch to more active modes would result in longer trip duration) [11]. The physical risks are most often associated with cycling. However, recent research suggests that the benefits outweigh these physical [24] and mental [11] risks. The overall benefit to society is larger as a switch to active travel would result in reductions in traffic collisions, air pollution, and greenhouse gases [24].

### 1.2. Active Travel and the Built Environment

The relationship between the built environment and travel mode choice has been studied exhaustively [25]. That study highlighted that walking is most associated land use diversity, intersection density, and the number of potential destinations within walking distance. This is also supported by a review from a medical journal on physical activity that found that transportation environments contribute to explaining why people are physically activity or not [26]. Saelens and Handy [27] report positive relationships between transportation and density, but Ewing and Cervero [25] found that the link with population and service density were weak once other variables were controlled for. However, it could be argued that as much of the literature reviewed above comes from Anglo-Saxon countries (e.g., USA, Canada, Australia, and United Kingdom) with a tendency to favor car use and single-use zoning, that the findings may be different if predominantly mixed land-use countries with better supporting transportation infrastructure (e.g., public transport, cycling lanes, *etc.*) were equally represented. Higher densities of population and services may also create congestion, which would diminish the utility of using motorized vehicles that require much more space [28,29] and are less flexible in their maneuverability than active modes. Further, greater population density is often required to support a greater diversity in local destinations that would support local non-motorized trips and higher public transportation service levels.

Empirical evidence in North America suggests that car travel is lower in traditional-style neighborhoods characterized by higher densities and a mixture of land uses where accessibility is often better with more pedestrian-orientated design features that encourage greater use of non-motorized modes (e.g., [25,30,31,32,33]). Similar results have been found in Europe [34,35,36] and Japan [37,38].

Such differences likely help explain the differences found for active travel. However, a recent study in China on middle-aged adults did not find a correlation between physical activity gained through transport and subjective environmental attributes [39], which may suggest that in areas where active travel is common, the built environment may play less of a role. However, Fishman, Böcker and Helbich [9] using objective measures found that higher densities were more associated with active travel in the Netherlands. Related to perceived environmental quality, a study in Japan found that for elderly perceptions of the social-environmental quality and aesthetic qualities were consistently positively associated with walking for transport and for leisure [40].

As described above, the built environment has been found to have an influence on transportation choices. One question that often arises is the question of self-selection where the argument is that it is not the built environment that has a significant influence on travel, but that the people who choose to locate in walkable neighborhoods are also the people who are pre-disposed (whether due to personal desire or some constraint) to travel by active modes. Early evidence did find that for the USA, attitudes and values were a greater explanatory variable than the built environment [41]. That question has subsequently been addressed, and the findings support the argument that the built environment does have an influence beyond that of personal attitudes and values (e.g., [42,43,44]).

Confounding the impact of the built environment are two key considerations: household car ownership and household life-cycle stage. With respect to studies on active travel, although living in more urbanized areas is associated with greater active travel, car ownership is also a strong negative influence [9]. Household car ownership has long been known to have an impact on mode choice in favor of car use [45,46] and can affect the results in at least two ways. The first relates to the negative effect of owning a car on the likelihood of making all trips by active travel or public transport [47,48,49]. The second is that a greater ownership level would act as a proxy for the value or preference put on car use by the household [50].

### 1.3. Lifecycle Stage and the Built Environment

The travel desires and needs of people will vary over their lifetime. Ewing and Cervero [51] in a review emphasized that sociodemographics explained more than the built environment for trip frequencies, that it combined with the built environment to explain mode choice, and that the built environment was primarily responsible for trip length. Along the same findings, Sun, Waygood, Fukui and Kitamura [37] found for Japan that when household lifecycle stage was considered (e.g., is the household a couple, a couple with young children, a couple with teenagers, *etc.*) the built environment explained the percentage of trips by cars for a household, but that it was the lifecycle stage that explained the number of trips. In line with this, Chatterjee *et al.* [52] also found that life-change events could lead to changes in bicycle use throughout an individual’s life course in the UK.

Amongst life-change events, a change in the household make-up from childless to having dependent children will impact travel needs and desires. A young, childless couple will behave differently from a family with children, who will in turn behave differently from a household with adult children, and so on [37,53]. Heggie [54] and Zimmerman [55] found that the lifecycle effect in travel was caused by two separate components: household structure and the age of household members. Zimmerman [47] argued that over the lifecycle, a household’s trip making will be determined by the relative contribution of these two separate components. The lifecycle classification variable is a composite one that subsumes various causal factors that combine to create different between-group patterns that can be observed [56].

Households at the same lifecycle stage will have similar demands on their time and the built environment likely affects how they travel. As mentioned, the lifecycle stage of a household related strongly to the number of trips conducted by the household [37]. For example, a household defined as ‘‘preschool nuclear’’ conducted roughly twelve trips per day, whereas a household defined as ‘‘all adults’’ conducted roughly eight trips per day. For both households though, less than 20% of trips were by automobiles in the two most developed built environments and roughly 40% or more in the least developed ones. That study, however, only examined the propensity to use automobiles for travel and did not distinguish other means of getting around such as active travel and public transport use, which would contribute to daily physical activity.

Thus, for this article the research questions are:

(1) Given car ownership and lifecycle stage of households, is the built environment associated with differences in active travel min? (1a) What is the share of cycling and walking min for public transport trips?
(2) Is the built environment associated with achieving the WHO recommendation of 10-min bouts of physical activity?
(3) Is the built environment associated with achieving the WHO recommendation of an accumulated 150 min over a week?

Based on the literature review, the hypotheses proposed are:

(1) More urbanized areas (*i.e.*, those with higher population and service densities) will be associated with greater active travel.
(2) This association will hold even taking into consideration household lifecycle stages (related to transport needs and desires) and car ownership (negative influence on using alternative modes such as active travel or public transport).

## 2. Experimental Section

### 2.1. Previous Work in Study Area

This paper builds on previous research on travel patterns and impacts from the Osaka Metropolitan Area (including the cities of Osaka, Kyoto, and Kobe). Previous research has examined the role of the built environment and life-cycle stages on car use and carbon dioxide (CO_2_) production.

The built environment was previously demonstrated to explain more than the lifecycle stage of a household for the percentage of trips by car, whereas the lifecycle stage explained the number of trips made [37]. With respect to transport CO_2_ emissions, the average energy use per km for motorized vehicles was examined in Waygood *et al.* [57], which included all modes. In that study, distances by non-motorized modes increase from the most urbanized to the least, and non-motorized modes represent a high of 59% in the most urbanized down to 25% in the towns (it was 32% in the rural/unurbanized). However, there has been a general spreading out of the built environment over the past decades [50] that is likely linked to more car-oriented development [58,59] resulting in increasing trip distances, which could lead to a lower active travel if those trips are made by motorized modes (though the contribution of active travel to public transport trips is not known). As well, no clear influence of car ownership was found to increase the number of trips (a proxy for activity engagement), but not the average duration of trips when the built environment and lifecycle stage were taken into consideration [60]. The influence of the car was more evident in less developed areas [60].

Although car use is increasing, many local trips such as for shopping during the week are still by active travel modes [58]. Further, most trips are not by car in this area [37], and children have very low car use, though it is again higher in the least urbanized areas [61]. In a study of children aged 10 and 11 years old in the OMA, Waygood and Kitamura [62] found that children gained on average 44 min of active travel in medium density neighborhoods (2000–3999 people/km^2^) and 27.3 min in the very high-density neighborhoods (over 6500 people/km^2^). The other two areas, low density and high density, had 41 min and 34 min, respectively. In that study, it was also found that the children reported more running-level (e.g., vigorous) activities in the lower density areas, but the differences were not significant.

Here, the research question relates to a societal measure of daily physical activity. Thus, it will further contribute to a more holistic understanding of the sustainability of these development types.

### 2.2. Data Source

**Figure 1:**
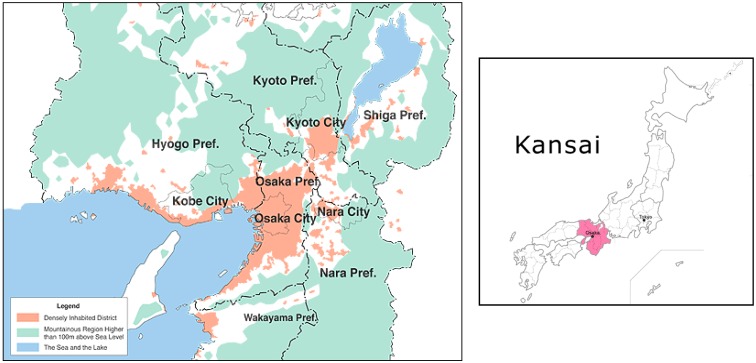
Study area is the Kei-Han-Shin area, which includes the Prefectures (Pref.) of Kyoto, Osaka, and Kobe.

For this analysis, the Osaka metropolitan area (OMA; Figure 1) person trip data for the year 2000 was used because it is supported by supplementary work on land-use [63], network data, and household lifecycle stages [37]. This was a conventional large-scale household travel survey with a sampling rate of 3.0%. This dataset contains the socio-demographic characteristics of the observed samples as well as their household characteristics. An adult entered information on children under the age of 15 years. The survey also includes the duration, purpose and number of activities, and trip engagements of the observed samples on the observed day. Further, the chosen travel modes, as well as home and work locations (zone) of the observed individuals were recorded.

The area, also known as Kei-Han-Shin, contained roughly 20 million people over 11,000 km^2^ of land. Considerable rail development occurred in the first half of the 20th century that enabled rail-based suburbanization. More recently (since after the 1970s), development has been more car-oriented with an associated greater tendency to use cars for trips (e.g., [37,58,64]). However, the tendency to commute from suburban locations to work by car found in other countries is perhaps diminished by the user-pays system for the highway network in Japan [65] and company-paid public transit commuter passes [66].

### 2.3. Household Lifecycle Stages

The lifecycle stages were developed primarily through analysis of household characteristics such as children’s age(s) and the age of the “head-of-household”. Ten distinct stages of lifecycle were formulated, as shown in Table 1.

**Table 1:** Descriptions and Definitions of lifecycle stages.

Household Lifecycle Stage	Descriptions	Definitions
Younger single	Younger single household	Single adult younger than 60
Younger childless couple	Younger childless-couple household	Oldest person younger than 60
Pre-school nuclear	Nuclear families with pre-school children	Youngest child younger than 6
Young school nuclear	Nuclear families with young school children	Youngest child 6 or older but younger than 12
Older school nuclear	Nuclear families with older school children	Youngest child 12 or older but younger than 18
All adults	Families of all adults	Nuclear families and single-parent families with all members of working age
Older childless couple	Older childless-couple household	Oldest person 60 or older
Older single	Older single household	Age 60 or older
Single parent	Single-parent household	Youngest child younger than 18
Others	Other households	Families with three generation, other related persons, and unrelated persons

### 2.4. Built Environment Categories

As opposed to using variables such as population density, service density, or the number of different types of shops, built environment categories are used in this study. Although those individual measures could be used, a problem of collinearity exists where a number of measures are closely correlated (such as population and service density). A built environment is composed of a mix of factors that can influence travel choices, thus the classification of built environments allows the research to capture essential differences between the types. The five built environment categories used in this research are those developed by Fukui [63], and their representative characteristics can be found in Table 2. The areas were estimated through cluster analysis of various factors such as information about the residences’ characteristics, the densities of both people (daytime and night) and shops, along with the employment situation (e.g., more jobs than residences or vice-versa; this suggests how much commuting for work would be necessary).

**Table 2:** Household and area characteristics of the five built environment areas.

Measure	Statistic	Highly Commercial	Mixed Commercial	Mixed Residential	Autonomous	Unurbanized
**Household Size**	Range	1 to 10	1 to 13	1 to 13	1 to 10	1 to 9
	Average	2.2	2.5	2.9	3.1	3.3
**HH Cars**	Range	0 to 12	0 to 11	0 to 15	0 to 12	0 to 15
	Average	0.49	0.623	1.11	1.63	1.82
**HH Motorcycles**	Range	0 to 10	0 to 8	0 to 11	0 to 7	0 to 6
	Average	0.15	0.17	0.3	0.45	0.5
**HH Bicycles**	Range	0 to 7	0 to 9	0 to 9	0 to 9	0 to 9
	Average	1.13	1.44	1.34	1.36	0.92
**Population Density (people/km^2^)**	Range	4224 to 12594	5493 to 18757	48 to 16114	74 to 2457	35 to 1976
	Average	8985 (36/acre)	12,620 (51/acre)	3770 (15/acre)	1138 (4.6/acre)	493 (2.0/acre)
**Service Density (businesses/km^2^)**	Range	222 to 1137	97 to 776	0 to 208	0.6 to 25	0.5 to 15
	Average	558 (2.3/acre)	189 (0.76/acre)	38.1 (0.15/acre)	15 (0.06/acre)	4 (0.02/acre)

**Figure 2:**
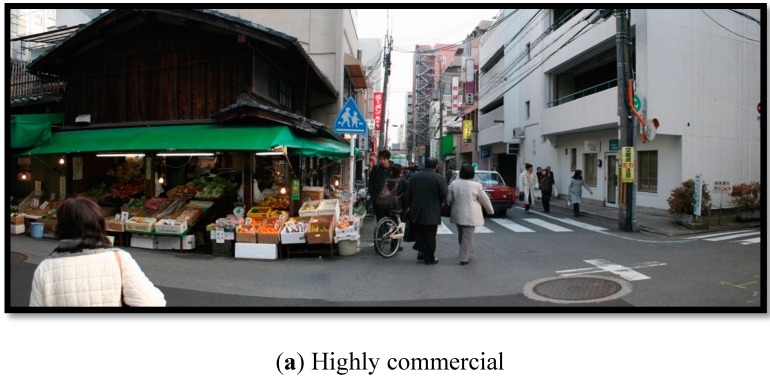

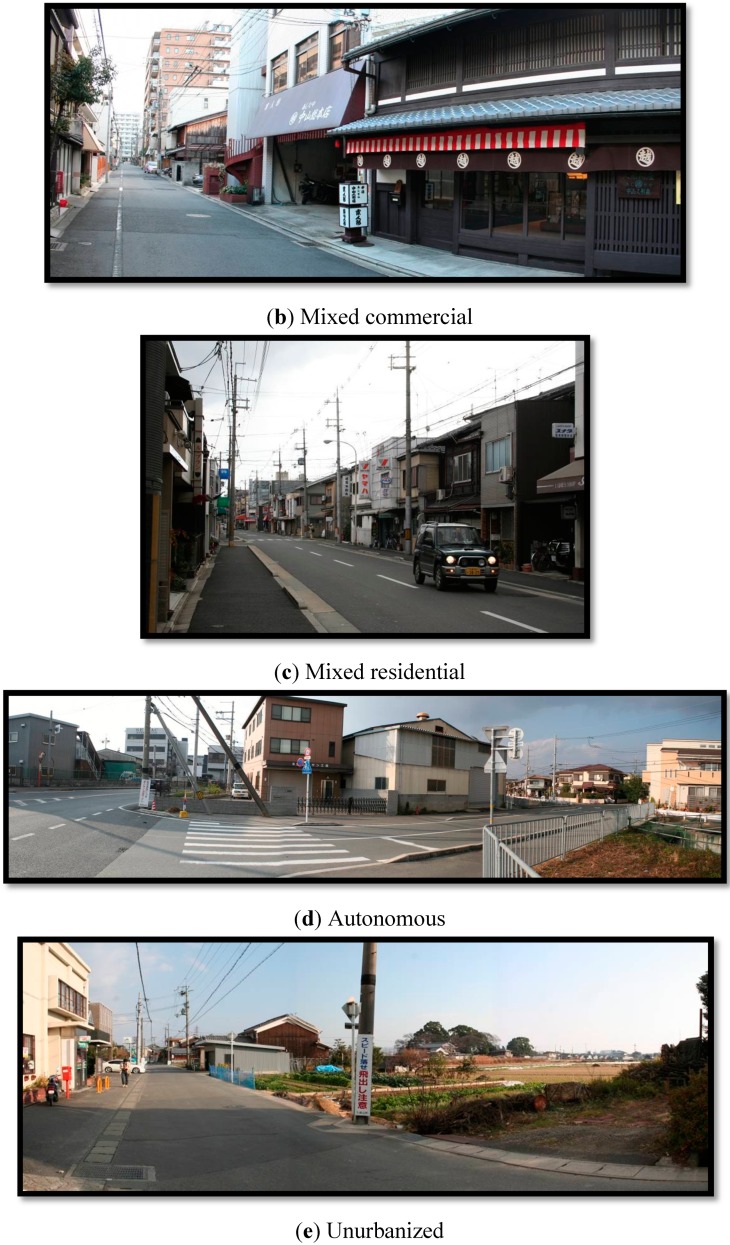
Representative images of the built environments in this study [67].

**Figure 3:**
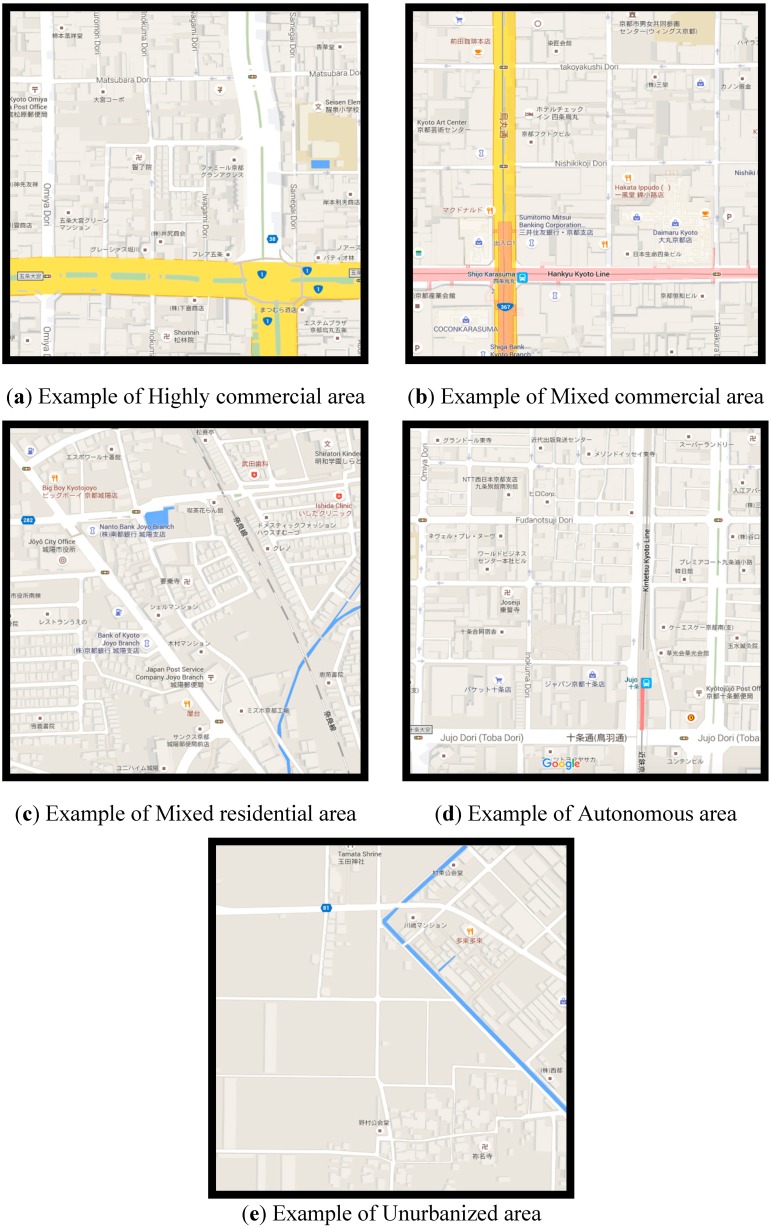
Map examples of the different area types (images from Google Maps).

Representative images are given in Figure 2 and representative maps in Figure 3. It should be noted that nearly all non-arterial roads are typically what would be referred to as “shared-space” in Western planning, as sidewalks are not common and traffic is a mix of pedestrians, cyclists, and motorized vehicles. The basic descriptions of each area are described as follows:

(1) A *Highly commercial area* is an area with the highest densities of commercial development (service densities 558/km^2^). These areas have a high daytime population with respect to the nighttime population. Such areas could be termed car-restrictive as roads are typically narrow apart from arterial roads. Pedestrian-only roads are commonly found in such areas.
(2) *Mixed commercial areas* have a high density of commercial development (service densities average 189/km^2^) and have high-density residential development as well (population densities average 12,620 people/km^2^). There is a less distinct change in the day- and nighttime populations. Such areas are also relatively car-restrictive as non-arterial roads are often narrow. Pedestrian-only roads are not as common but can be found.
(3) *Mixed residential areas* do not have sufficient work for the population (service densities average 38/km^2^) and some residents must commute to another area. These areas are distinguished by a higher nighttime population than daytime. However, these areas can have high population densities (up to 16,114 people/km^2^) that support high store and service densities (up to 208/km^2^). They typically represent the growth regions of the main urban areas. Narrow roads are common.
(4) *Autonomous areas* have a roughly equal amount of residential and commercial development that allows residents to live and work within the area. There is not significant change in the day- and nighttime populations. However, the population density is lower (average 1138 people/km^2^) and these areas are located separate from major urban centers. These are typically distinct towns from the main urbanized area. These areas often include more car-oriented development with larger roads and shops with parking in front.
(5) *Unurbanized* have low densities of both commercial (average business densities 4/km^2^) and residential (average people densities 493/km^2^) development and low employment opportunities. These are typically agricultural areas. Roads are not typically large here, but due to the low population densities, distances between locations is often large and traffic levels are low.

### 2.5. Descriptive Trip Information

**Figure 4:**
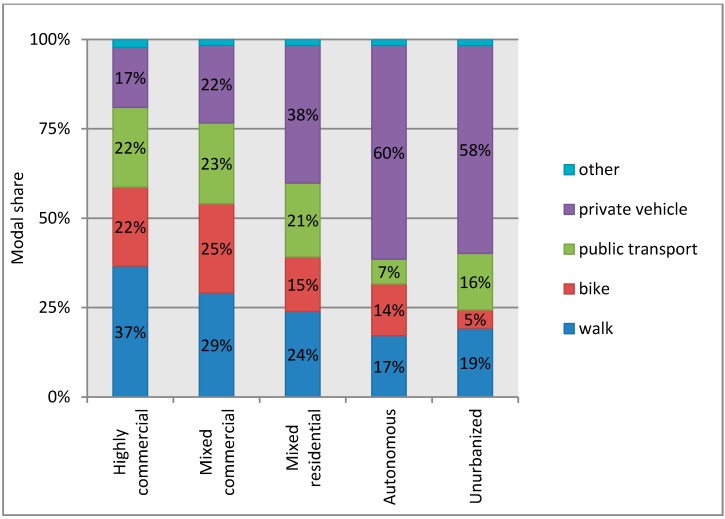
Modal share by built environment type.

Before presenting the results of the statistical models, several descriptive results are presented here. The first shows the modal share by built environment (Figure 4). The modal share is the percentage of all recorded trips by the different means of travel. Travel by moped (50 cc or less), motorcycle, subcompact car (<660 cc), passenger car, and trucks/vans are combined into *private vehicle* trips. Travel by bus and rail are combined for *public transport* trips. *Other* contains trips by taxi, charter bus, and other infrequent modes. A hierarchy of modes is applied where trips that contain more than one mode are classified in this order: airplane, boat, rail, bus, charter bus, passenger car, truck/van, subcompact car, taxi, motorcycle, moped, bicycle, other, wheelchair, and finally walking. As can be seen, the modal share of active travel for the two most urbanized areas is greater than 50% and public transport over 20%. For the next most urbanized (Mixed residential) area, active travel represents nearly 40% and public transport is again roughly 20%. In the two less urbanized areas, active travel represents about 30% (Autonomous) and 25% (Unurbanized) with public transport representing less than 10% and roughly 15%, respectively.

**Figure 5:**
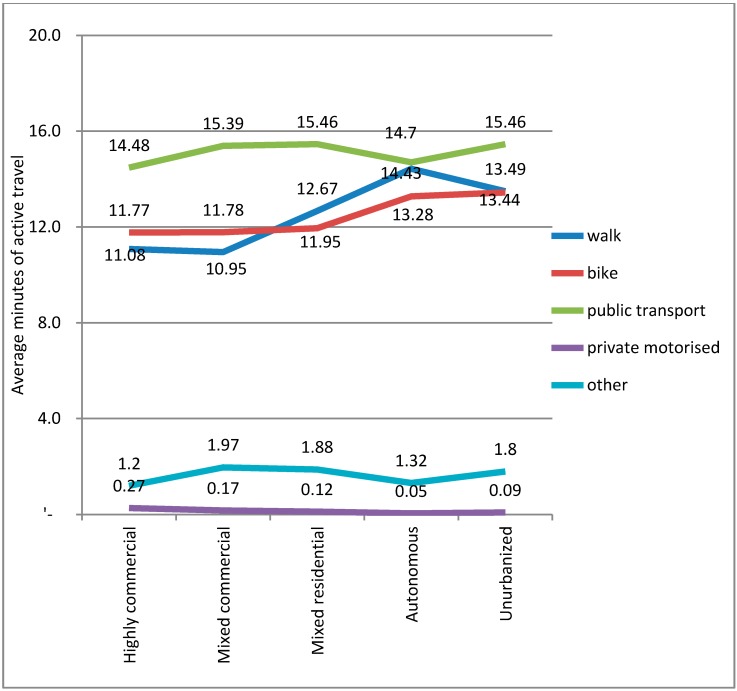
The average active travel time per mode by built environment type.

As the interest here is active travel by mode, the average active travel time per mode by built environment type is shown in Figure 5. The data used in this study contains the min travelled by each mode during a trip. Thus, for example, public transport trips include the access time (typically by active modes) and the duration of the final leg to the destination (again typically by active modes). Here, it can be seen that people are walking or cycling for longer durations with trips associated to public transport in all of the built environment types. Although not shown here, the majority of these minutes are associated with walking (that is people are mostly walking to access public transport). The percentage of walking trips associated with bus trips was from 92.1% in the Unurbanized areas up to 98.4% in the Highly commercial areas. For rail it is slightly lower, with a low of 73.8% in the Autonomous areas, 88.3% in the Unurbanized, 86% in the Mixed areas, and 93% in the Highly commercial. Those results are intuitive as bus stops are typically closer and bicycles are competitive with the bus for access. Rail stations are typically spaced farther apart and thus the cycling percentage increases for those longer distances. Next, one observes that the average duration of active travel increases between the more urbanized and less urbanized areas. This follows the logic that in more urbanized areas, destinations will be closer (*i.e.*, shorter distances) as a result of higher population and service densities, thus trip durations are shorter.

**Figure 6:**
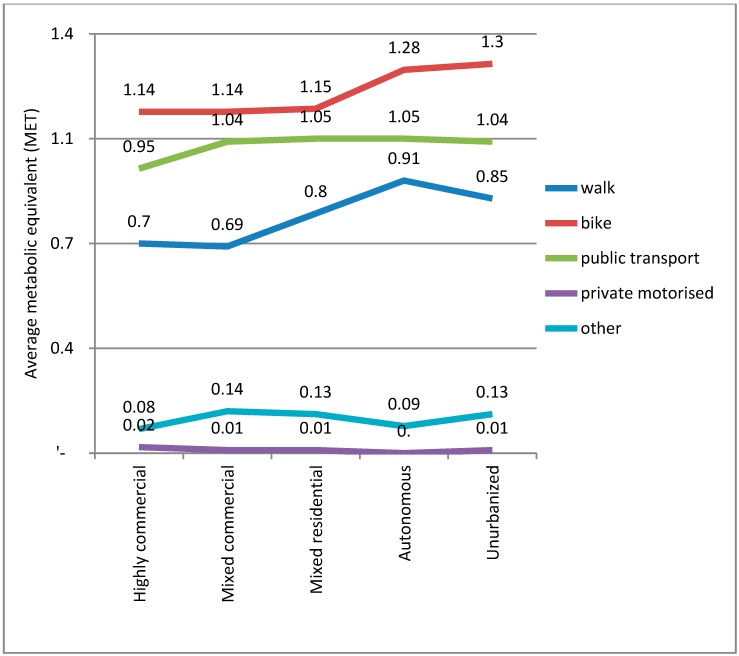
The average MET hours by mode and built environment type.

Applying the metabolic equivalents of tasks (METs = 1 kcal/kg/h) that were used by Fishman, Böcker and Helbich [9], this would suggest that cycling produces the greatest benefit per trip, followed by public transport, and then walking. The other modes are negligible in comparison (Figure 6).

**Figure 7:**
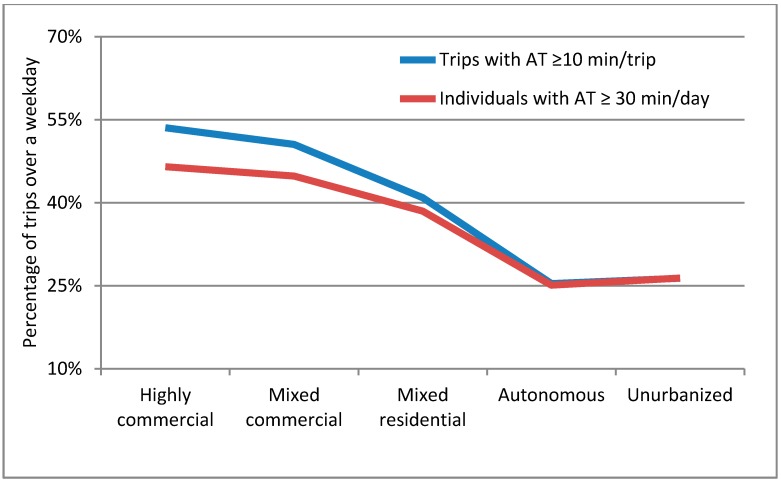
Percentage of trips by built environment where active travel min are at least 10 min and the percentage of respondents achieving at least 30 min accumulated active travel min by built environment type.

Next, considering the recommendations that physical activity doses should be at least 10 min [2] and that adults should achieve an accumulated total of 150 min per week, the percentage of individuals achieving those thresholds by built environment type are shown in Figure 7. As the survey is a one-day trip dairy on a weekday, we assume that each weekday is roughly equivalent so that one would need 30 min per weekday to achieve the 150 accumulated physical activity min threshold. As can be seen, in all of the built environments, at least 25% of the respondents achieve these two thresholds. As urbanization intensifies, this figure increases to a maximum of 53.5% of trips achieving a minimum of 10 min of active travel and 46.5% reaching the 30 accumulated min per day in the Highly commercial area. The differences between the gains from active travel for public transport to those associated with private motor are in the most common range (12–15 min) reported in previous studies [6].

## 3. Results and Discussion

### 3.1. Built Environment, Household Lifecycle Stage, and Car Ownership

**Figure 8:**
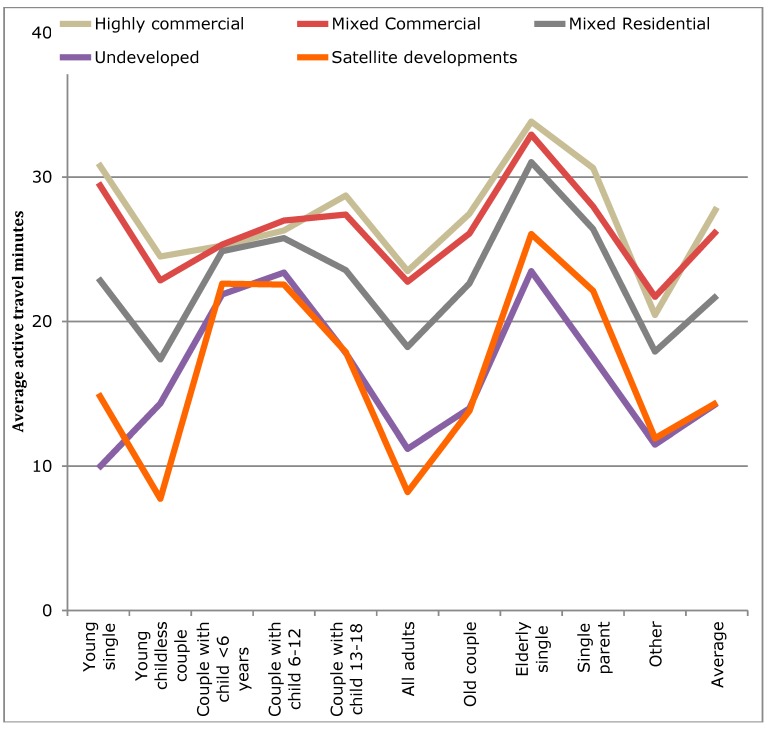
Changes in average active transport time per person for each built environment by lifecycle stage.

**Figure 9:**
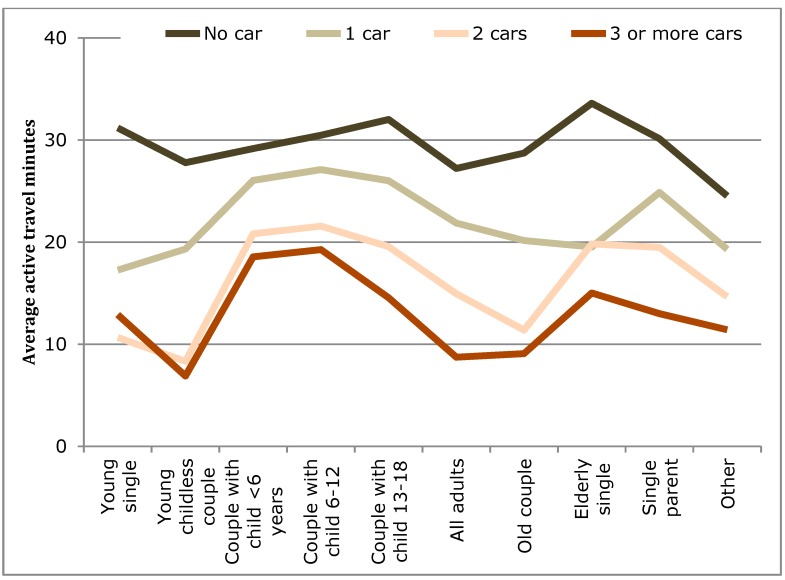
Changes in average active transport time per person for car ownership by lifecycle stage.

**Figure 10:**
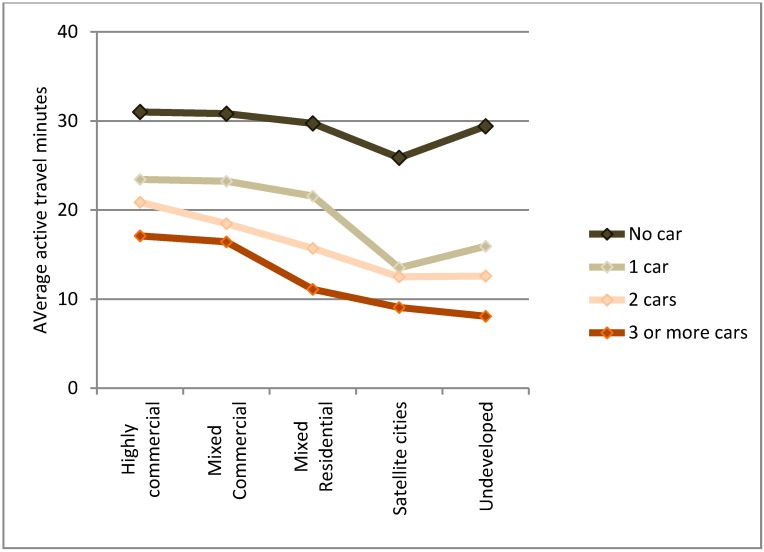
Changes in average active transport time per person for car ownership by built environment type.

Analysis of variance, ANOVA, was carried out on the total accumulated active travel (here, walking and cycling trips, and any such trips recorded in relation to public transit use) over a day by car ownership, lifecycle stage (LCS), and built environment (BE) (Table 3). The average min of active travel per day are shown in Figure 8, Figure 9 and Figure 10 for car ownership by BE, car ownership by LCS, and BE by LCS, respectively.

**Table 3:** ANOVA analysis of *total household trips* by car ownership, lifecycle stage (LCS), and built environment (BE).

Source	Type III Sum of Squares	df	Mean Square	F	Sig.
**Corrected Model**	5.455E6	194	28,117	73.6	0.000
**Intercept**	636,403	1	636,403	1666.6	0.000
**Built Environment (BE)**	26,285	4	6571	17.2	0.000
**Life-cycle stage (LCS)**	27,874	9	3097	8.1	0.000
**HH car ownership (HH_CAR)**	31,261	3	10,420	27.4	0.000
**LCS × HH_CAR**	36,229	27	1342	3.5	0.000
**BE × HH_CAR**	20,316	12	1693	4.4	0.000
**BE × LCS**	23,470	36	652	1.7	0.005
**BE × LCS × HH_CAR**	44,398	103	431	1.1	0.175
**Error**	4.201E7	110,001	382		
**Total**	1.035E8	110,196			
**Corrected Total**	4.746E7	110,195			

As can be seen in Table 3, all variables and interactions are significant except for the three-way interaction. The results suggest that car ownership is the strongest influence as the largest sum of squares results were for the three-way interaction, then the LCS and car ownership interaction, followed by the single influence of car ownership level. However, all three influences (car ownership, built environment type, and household lifecycle stage) had comparable singular influences. As can be seen in Figure 10, the step changes between the built environment types are smaller than those for increase car ownership. However, if a distinction were simply made between urban (Highly commercial, Mixed commercial, and Mixed residential) and other (Autonomous and Unurbanized), the step change for built environment would increase. Finally, visually it can be seen that large variation occurs by household lifecycle stage (Figure 8 and Figure 9), demonstrating that it is important to take into account such factors when examining travel behavior. 

### 3.2. Achieving the WHO Recommended Physical Activity Thresholds

#### 3.2.1. Physical Activity in a Minimum of 10 min Bouts

As described above, the WHO’s recommendations for physical activity require that the physical activity bout is at least 10 min. Thus, here, the likelihood that the active travel portion of a trip achieves that threshold (*i.e.*, active travel minutes ≥ 10 min) is analyzed through binary logistic regression (Table 4). As can be seen in the second model, living in a Mixed commercial or Mixed residential areas increased the likelihood (1.5 and 1.3 times) of achieving this threshold as compared to living in a Highly commercial area, while living in the less urbanized areas decreases that likelihood (1.3 and 1.2 times less likely). Car ownership had a stronger impact, as with each additional car a household owns there is roughly a further two times less likelihood of achieving that recommendation as compared to households without cars (car free).

**Table 4:** Binary logistic regression results for the active travel portion of a trip being equivalent or greater to 10 min (*n* = 1,027,622). Reference variables are shown by (ref.).

Higher Order Grouping	Variable	Odds Ratio (1st)	Odds Ratio (2nd)
	Constant	1.379	0.983 * (*p* = 0.1)
Individual characteristics	Child	Not included	4.96
	Male	0.856 (−1.17)	0.8 (−1.25)
Built environment (reference variable is Highly commercial areas)	Highly commercial (ref.)	1.0	1.0
	Mixed commercial	1.463	1.532
	Mixed residential	1.245	1.268
	Autonomous	0.869 (−1.15)	0.764 (−1.31)
	Unurbanized	0.794 (−1.26)	0.843 (−1.19)
Household car ownership (reference variable is Car free)	Car free (ref.)	1.0	1.0
	1 car	0.474 (−2.11)	0.455 (−2.20)
	2 cars	0.275 (−3.64)	0.249 (−4.01)
	3 cars or more	0.179 (−5.59)	0.162 (−6.16)
Household life-cycle stages (reference variable is Couple with child 6–12)	Single and <60	0.676 (−1.49)	0.919 (−1.09)
	Couple < 60	0.738 (−1.36)	1.096
	Couple with child <6	1.181	0.807 (−1.24)
	Couple with child 6–12 (ref.)	1.0	1.0
	Couple with child 13–18 years	1.456	1.332
	All adults	1.07	1.614
	Couple > 59	0.812 (−1.23)	1.189
	Single > 59	0.862 (−1.16)	1.213
	Single parent	1.197	0.757 (−1.32)
	Other	1.187	1.364

* Not significant at *p* < 0.05; 1st: Cox and Snell *R* square = 0.069; Nagelkerke *R* Square = 0.093; 2nd: Cox and Snell *R* square = 0.131; Nagelkerke *R* Square = 0.176.

Children are much more likely to achieve this goal (nearly five times) than adults. Effectively, the difference between the two models is comparing the child’s age at the different stages and adult behavior between the lifecycles. So, for example, the lifecycle stage “all adults” is nearly equivalent to a household with children aged six to twelve in the first model, but is 1.6 times more likely to conduct active travel trips of at least 10 min when children’s trips are accounted for. For single parents, one can see a reverse in the influence between the two models, which would suggest that children in single parent households are active, but their parents are less active than a household with two parents.

Using the complete model (the one including a child variable), as compared to belonging to households with children between the ages of six and twelve, being single and under 60 years old (9% less), having a child less than six years old (24% less), or being a single parent (32% less) were associated with a lower likelihood of achieving that threshold. The remaining household lifecycle stages were associated with greater likelihoods of achieving that recommendation.

#### 3.2.2. A Weekly Accumulation of 150 min

The WHO recommends that adults achieve 150 min of moderate physical activity over a week. As the results presented here relate to weekday trips, we assume that each weekday (*i.e.*, Monday through Friday) will have roughly the same activities, thus if an individual accumulates 30 min over a day, then one could infer that over five days this would amass to 150 min. The results of a binary logistic regression on an individual accumulating 30 min of physical activity through active travel is shown in Table 5.

**Table 5:** Binary logistic regression results for an individual achieving an accumulated 30 min of physical activity through active travel (*n* = 349,957).

Higher Order Grouping	Independent Variables	Coef.	Odds Ratio
	Constant	−0.017	0.983
Individual characteristics	Child	1.399	4.052
	Male	−0.204	0.815 (−1.22)
Built environment (reference variable is Highly commercial areas)	Highly commercial	(ref.)	1.0
	Mixed commercial	0.339	1.404
	Mixed residential	0.242	1.274
	Autonomous	−0.193	0.825 (−1.21)
	Unurbanized	−0.093	0.911 (−1.10)
Household car ownership (reference variable is Car free)	Car free	(ref.)	1.0
	1 car	−0.606	0.546 (−1.83)
	2 cars	−1.122	0.325 (−3.07)
	3 cars or more	−1.524	0.218 (−4.59)
Household life-cycle stages (reference variable is Couple with child 6–12)	Single and <60	−0.054	0.948 (−1.06)
	Couple < 60 (*p* = 0.48) *	−0.012	0.988
	Couple with child <6	−0.142	0.867 (−1.15)
	Couple with child 6–12	(ref.)	1.0
	Couple with child 13–18 years	0.18	1.197
	All adults	0.274	1.315
	Couple > 59	0.079	1.082
	Single > 59 (*p* = 0.67) *	0.01	1.01
	Single parent	−0.385	0.68 (−1.47)
	Other	0.172	1.187

* Not significant at *p* < 0.05; Cox and Snell *R* square = 0.102; Nagelkerke *R* Square = 0.139.

Similar to the findings for a trip containing 10 min or more of active travel, living in a Mixed commercial or Mixed residential area increases the likelihood of accumulating 30 min of active travel over a day, where as living in the less urbanized areas decreases that likelihood as compared to living in a Highly-Commercial area. Again, car ownership is strongly associated with decreases in the likelihood of this health benefit being achieved (−1.8 times for 1 car, −3.1 times for 2 cars, and −4.6 times for 3 or more cars). Being under 20 years increases the probability by over four times as compared to being an adult (the WHO recommendation is for 60 min per day for children and youth). Males are less likely (−1.2 times) than females. As with the previous findings, living in one of three household lifecycle stages are associated with a decreased likelihood as compared to households that are composed of a couple with their youngest child being between the ages of six and twelve: single and under 60 years of age (−1.1 times); couple with a child under five years of age (−1.15 times); and single parent (−1.5 times). The remaining had positive influences as opposed to the reference variable of couple with a child between the ages of six and twelve.

## 4. Discussion

The results presented in this paper support the hypothesis that active travel is more commonly practiced in more urbanized areas (e.g., [9,25]). Car ownership has a greater individual association with a decrease in active travel min than the built environment categories, though the differences between the urban centers and less urbanized areas is similar in magnitude of change. Differences were found by lifecycle stage, suggesting that the household needs and desires will influence how much active travel is gained. However, as hypothesized, the built environment continued to have an association with active travel even when car ownership and household lifecycle stage were accounted for. The findings also suggest that considerable active travel gains are associated with public transport, and that the majority of those min are through walking.

The results of the binary logistic regression show that when car ownership and household lifecycle stages are accounted for, there is a clear impact of the built environment on the likelihood that a trip will contain at least 10 min of active travel and that an individual will accumulate at least 30 min of active travel over a day. This is similar to Fishman, Böcker and Helbich [9] who used a continuous variable for car ownership and found a strong negative influence on the total METs achieved through active transport over a day.

Within the more urbanized areas (those typically related to the larger cities within the metropolitan area) the association between greater average active travel min and the built environment was not linear. Examining simply the averages (Figure 8), it would appear that the largest average min of active travel per person was a step progression away from the most urbanized area (*i.e.*, the Highly-commercial areas) to the least (*i.e.*, Unurbanized). However, in the binary regression models on the WHO recommended targets (Table 4 and Table 5), when the household lifecycle stage, car ownership, and individual factors (child, male) were accounted for, it was found that the order was: Mixed commercial, Mixed residential, Highly commercial, Unurbanized, and finally Autonomous. This is likely explained by a combination of two key factors that interact. The Mixed commercial area has maintained low car use over the past several decades [37], but the distances to locations is slightly greater than the most urbanized areas. The Mixed residential areas, though having higher car use [37], also benefit from slightly longer distances, but distances that are still in favor of active travel. The least urbanized areas (Autonomous and Unurbanized) have experienced the greatest car use increases [37], and the development style of Autonomous areas is more car-oriented. In the Unurbanized areas, large distances decrease the likelihood of active travel.

In the study area, it was further noted that even in the less urbanized areas at least 25% of population is possibly reaching the recommended level of physical activity solely through their transport, rising to 47% and 45% in the two most urbanized areas. Taking the population by built environment type, it can be calculated at 40% of the population is reaching 30 min per day of active travel, which is similar to the 38% reported by Fishman, Böcker and Helbich [9] for the Netherlands.

From the descriptive results, it is apparent that when considering active travel it is important to also include the min associated with public transport trips. Although not shown above, the variation for bus and rail by built environment type was not great with the average active travel for buses being 11.5 min (±0.7) and rail-based transit 15.5 min (±0.4). Active travel for walking trips was 12.5 min (±1.5) and for cycling trips it was 12.4 (±0.8). It should be noted that research suggests that such trips are generally underestimated [68].

The findings suggest that car ownership is a stronger explanatory variable than the five built environment types. If the averages of active travel minutes by built environment type are compared, the differences in minutes of active travel between the most urbanized area (Highly commercial; 27.9 min) and each successive decrease in urbanization are respectively: −1.6 min (MC), −6.1 min (MR), −13.5 min (A), and −13.6 min (U). The largest step change is thus from Mixed residential (MR) area to the Autonomous (A). The latter was previously found to have the highest car use in the study area [37]. Thus, it can be seen that a clear difference exists between the more urbanized areas and the two less developed areas. The distinctions likely relate to population and service densities, but also higher levels of service for public transit. The population density supports the service density in a mixed land-use planning system (see [69]), while the public transit service levels support commuting and longer distance travel. The smallest impacts of car ownership on active travel minutes was observed in the two most urbanized areas (Highly and Mixed commercial).

The differences for car ownership from no car ownership (30.1 min) were: −8.55 min (1 car), −14.52 min (2 cars), and −19.11 min (3 or more cars). The impact of the car is likely related to personal preferences, so an individual who is less inclined (or less able) to take modes that would include active travel (including public transit) would be more likely to purchase a car. Thus, there is likely a combined effect of owning a car, which would reduce other mode use and an individual’s general likelihood to use those other modes. However, car ownership is related to household location with higher ownership in areas that are less urbanized [37] or farther from the main three cities of Kyoto, Kobe, and Osaka [70]. If car ownership is to be limited, individuals require basic needs and services to be (easily) accessible by either active travel or public transport. That generally requires mixed land-use and a population density that can support local shops. Thus, although car ownership was a stronger explanatory variable, the built environment is a key means of supporting car free or low car ownership households.

In less dense areas (such as the Autonomous and Urbanized areas in this study), where being car-free is next to impossible, car-sharing programs might be part of the solution, maintaining flexibility and access to services while reducing car ownership. At the time of the data collection, such systems had not yet been introduced to the study area, so its impact cannot be seen here. Research has shown that car sharing programs lead to decreased car ownership and increased active travel [71,72]. The suggested explanation would be that when faced with the true costs of trips (costs of car-share trips usually involve a mileage fee), people tend to choose a cost effective mode of transportation (*i.e.*, walk or cycle for short trips and bus when available).

The household lifecycle stage (LCS) was found to influence active travel in a number of ways. When average AT minutes per household individual were examined (Table 3) significant differences were found between the LCSs (Figure 8). The peaks here were found for the elderly, single parents, young singles, and households with children. However, when children were accounted for in the analyses for the recommended measures of 10-min bouts (Table 4 and Table 5) and an accumulation of 30 min over a day, it was found children are achieving these targets, but they appear to have a negative influence on their parents. In fact, the greatest difference can be seen on single parents.

Other influences were examined as well. Women were found to be more likely than men to do 10-min bouts of active travel (25% more likely) and to accumulate 30 min of active travel (22% more likely). This was in-line with the findings by Fisherman *et al.* (2015), but differed to findings in the U.S. [73] and in Australia [74]. Children were found here to be much more likely than adults to achieve 10-min bouts (5 times) and accumulate 30 min (4 times). Previous studies focused on adults, so it is not possible to make a comparison, though as previously shown by Waygood [75] and Susilo and Waygood [61] children in this area do not use private transportation often during the week.

The results of the household lifecycle stage suggest that the children’s age is also an important consideration. Many studies simply contain a binary variable for the presence of a child without consideration to the differences between an infant, a child who is beginning to have autonomy (e.g., able to travel without an adult), and an autonomous child. The results of this paper (in particular Table 4) suggest that increasing children’s autonomy could increase parents’ active travel.

The World Health Organization’s (WHO) Health economic assessment tool (HEAT) [76] can be used to measure the impact of active travel for a reduction in mortality risk (and economic impact as well, though not included here). The tool works on a dose-response relationship and takes into consideration both positive impacts (e.g., physical activity) and negative consequences (e.g., breathing in air pollution and potential road traffic collisions) [77]. Evidence for morbidity is more limited, so a mortality measure (the risk of death) is applied which likely results in a more conservative estimation of the benefits that are disease-related Morbidity would include benefits such as mental health, energy balance, and musculosketal health, however the evidence is currently insufficient so the WHO HEAT tool only calculates the reduction in mortality risk. 

The reductions in the risk of mortality to the population for this study are shown by built environment (Table 6) and by car ownership (Table 7) for each lifecycle stage. This is calculated using the HEAT online tool for walking [78] based on the daily duration of active travel. This appears to be a more conservative estimate as the numbers were estimated using the HEAT online tool for cycling assuming only travel on weekdays, and the reduction in the risk of mortality is slightly increased. As can be seen, the average reductions for the urban areas *versus* the more car-oriented areas are similar to the average reductions for no car ownership and two-car ownership. The average active transport by households with three or more cars shows the lowest reduction. To put this in perspective, the WHO found that for developed countries, the mortality risk factor of overweight in 2000 was 9.6% and 11.5% for men and women respectively (WHO, [79]). In the entire list only a few attributes had higher mortality risk factors: blood pressure (20.1% men and 23.9% women), tobacco (26.3% men and 9.3% women), and cholesterol (14.5% men and 17.6% women). Physical inactivity was 6.0% and 6.7% for men and women, respectively. All of the remaining attributes were much smaller (apart from alcohol for men which was 8%).

**Table 6:** Reduction in the risk of mortality to the population shown by built environment and lifecycle stage.

Household Lifecycle Stage	Highly Commercial Pop. 446,214	Mixed Commercial Pop. 5,236,070	Mixed Residential Pop. 11,204,440	Autonomous Pop. 1,136,635	Unurbanized Pop. 141,000
**Young single**	14.00	14.00	11.00	7.00	5.00
**Young childless couple**	11.00	10.00	8.00	3.00	7.00
**Couple with child <6 years**	12.00	12.00	11.00	10.00	10.00
**Couple with child 6–10**	12.00	12.00	12.00	10.00	11.00
**Couple with child 11–20**	13.00	13.00	11.00	8.00	8.00
**All adults**	11.00	10.00	8.00	4.00	5.00
**Old couple**	13.00	12.00	10.00	6.00	6.00
**Elderly single**	16.00	15.00	14.00	12.00	11.00
**Single parent**	14.00	13.00	12.00	10.00	8.00
**Other**	9.00	10.00	8.00	5.00	5.00
**Average**	13.00	12.00	10.00	7.00	7.00

**Table 7:** Reduction in the risk of mortality shown to the population by car ownership and lifecycle stage.

Household Lifecycle Stage	No Car	1 Car	2 Cars	3 or More Cars
**Young single**	14.00	7.00	5.00	5.00
**Young childless couple**	13.00	8.00	4.00	3.00
**Couple with child <6 years**	13.00	12.00	9.00	8.00
**Couple with child 6–10**	14.00	12.00	9.00	8.00
**Couple with child 11–20**	14.00	12.00	8.00	7.00
**All adults**	12.00	10.00	7.00	4.00
**Old couple**	13.00	8.00	5.00	4.00
**Elderly single**	15.00	8.00	8.00	7.00
**Single parent**	14.00	11.00	8.00	5.00
**Other**	11.00	8.00	7.00	5.00
**Average**	14.00	9.00	7.00	5.00

The Health economic assessment tool (HEAT) can be used to aid in cost-benefit analysis [78]. As an example of a financial impact of a switch from car use to cycling for a 5 km trip, Rabl and De Nazelle [80] found that the physical health benefits to the individual would be 1300 euros/year, and in a large city the reduction in air pollution would be an additional 30 euros/year. The negative impact of increased inhalation of air pollution would be roughly 20 euros/year, and the risk of collision varies significantly by context, but estimations are that the impacts would be an order of magnitude smaller than the benefits. For countries where cycling is common such as the Netherlands and Denmark, there appears to be an effect of either safety in numbers or general familiarity by drivers to cyclists [80]. Cycling in Japan is not associated with the majority of crashes, and even for youth where it is associated with more crashes, motorized modes have a greater association to fatalities [75,81].

Related to the household type (e.g., lifecycle stage), one observation can be made from Figure 8 and Figure 9. Children and elderly were more likely to walk than adults. This is consistent with published work on children’s travel in Japan where nearly all elementary-aged children walk to school [62] and are generally independent of parents [82]. For children in middle and high school years, a combination of walking, cycling, and public transport use is the most common [61].

Mental health benefits are more difficult to determine at this point. Active commuting and public transit use were found to have significant associations with overall psychological well-being [11]. Further, subjective well-being as a measure for how individuals evaluate their lives [83] finds that social relationships are an important explanatory variable and it has been hypothesized that walking contributes to building relationships and social capital, and recent findings suggest that at least for children this may be true. Thus, the benefits of active travel may not be fully appreciated in tools such as the WHO’s HEAT and more research is required to substantiate such potential advantages.

This paper did not examine the overall physical activity (PA) that individuals are achieving. In some research out of Asia, PA from transportation was not associated with environmental correlates for middle-aged adults [39], but more urban areas were associated with greater overall PA. In the same area of Japan as this study effort, the number of vigorous physical activities recorded with a travel dairy did not significantly vary over four built environment types of central city, mixed-residential, town, and rural [62]. That paper found that children in the less urbanized areas were on average gaining more physical activity through active travel with the explanation given as all children walk to school, children in less urbanized areas will walk for longer distances.

For trip duration, data from GPS systems would be more accurate, but it is unlikely that one could achieve sufficient information for a representative population that would allow for the analysis done here (five built environments × 11 life cycle stages × multiple car ownership levels). However, studies with GPS systems that show the biases can help with interpretation. For example, [84] found that nearly about 60% of trips are accurately reported with ±2 min should by in a household travel survey as compared to GPS results. About 20% were off by more than ±5 min. The majority (52%) of errors estimated longer durations *versus* 27% under-estimation. That study did suggest that the duration of car trips was over-estimated as compared with walking trips which would suggest that for this study, the results are likely fairly accurate.

## 5. Conclusions

This research examined the question of whether the built environment was associated with greater active travel when household lifecycle stages and car ownership were considered. The findings show that larger urbanized centers are more associated with active transport, that minutes gained through active travel related to public transport trips is significant, and that a large portion of the population is reaching the WHO’s recommendation of bouts of physical activity that last a minimum of 10 min and an accumulation of 150 min per week for adults. The household life-cycle was found to influence the average amount of active travel. In the binary regression analysis, where the built environment, car ownership, being a child or a male were accounted for, it became apparent that for the Japanese case, children are traveling by active modes, but their parents, as compared to other adults, are often less likely to meet the WHO recommendations. Car ownership was strongly associated with a decrease in active travel minutes and reaching the WHO recommendations.
